# Cellular and Molecular Phenotypes of pConsensus Peptide (pCons) Induced CD8^+^ and CD4^+^ Regulatory T Cells in Lupus

**DOI:** 10.3389/fimmu.2021.718359

**Published:** 2021-11-19

**Authors:** Ram P. Singh, Bevra H. Hahn, David S. Bischoff

**Affiliations:** ^1^ Research Service, Veteran Administration Greater Los Angeles Healthcare System, Los Angeles, CA, United States; ^2^ Division of Rheumatology, Department of Medicine, University of California, Los Angeles, Los Angeles, CA, United States; ^3^ Department of Medicine, University of California, Los Angeles, Los Angeles, CA, United States

**Keywords:** pCons, regulatory T cells, systemic lupus erythematosus, anti-DNA Ab, immune tolerance, immune regulation

## Abstract

Systemic lupus erythematosus (SLE) is a chronic autoimmune disease with widespread inflammation, immune dysregulation, and is associated with the generation of destructive anti-DNA autoantibodies. We have shown previously the immune modulatory properties of pCons peptide in the induction of both CD4^+^ and CD8^+^ regulatory T cells which can in turn suppress development of the autoimmune disease in (NZB/NZW) F1 (BWF1) mice, an established model of lupus. In the present study, we add novel protein information and further demonstrate the molecular and cellular phenotypes of pCons-induced CD4^+^ and CD8^+^ T_reg_ subsets. Flow cytometry analyses revealed that pCons induced CD8^+^ T_reg_ cells with the following cell surface molecules: CD25^high^CD28^high and low^ subsets (shown earlier), CD62L^high^, CD122^low^, PD1^low^, CTLA4^low^, CCR7^low^ and 41BB^high^. Quantitative real-time PCR (qRT-PCR) gene expression analyses revealed that pCons-induced CD8^+^ T_reg_ cells downregulated the following several genes: Regulator of G protein signaling (*RGS2), RGS16, RGS17*, *BAX, GPT2, PDE3b, GADD45β and* programmed cell death *1 (PD1).* Further, we confirmed the down regulation of these genes by Western blot analyses at the protein level. To our translational significance, we showed herein that pCons significantly increased the percentage of CD8^+^FoxP3^+^ T cells and further increased the mean fluorescence intensity (MFI) of FoxP3 when healthy peripheral blood mononuclear cells (PBMCs) are treated with pCons (10 μg/ml, for 24-48 hours). In addition, we found that pCons reduced apoptosis in CD4^+^ and CD8^+^ T cells and B220^+^ B cells of BWF1 lupus mice. These data suggest that pCons stimulates cellular, immunological, and molecular changes in regulatory T cells which in turn protect against SLE autoimmunity.

## Introduction

SLE is an autoimmune disease characterized by widespread inflammation, autoantibody production, and immune complex deposition. Regulatory T cells (T_reg_) are protective in many inflammatory and autoimmune diseases including SLE. The modulation of abnormal immune regulation is an object of intense investigation in autoimmune diseases. A therapeutic goal is to limit the number and activity of abnormal pathogenic cells and autoantibodies through restoration of immune system self-tolerance. One way to achieve that is by administrating peptides (such as pConsensus peptide, edratide and nucleosomal peptides) that induce regulatory T cells (1–11). Another approach used recently utilized nanoparticles for expanding regulatory T cells to treat autoimmune diseases including lupus (12–15). Whereas a decrease in the number and/or function of regulatory CD4^+^ T cells has been extensively studied in SLE (16–24), the role and characterization of the CD8^+^ T_reg_ subset is less clear. Investigating the genes, regulatory networks, and signaling pathways that regulate the functional activity and survival of CD8^+^ T_reg_ cells is important for development of therapies for restoring immune homeostasis in SLE and other autoimmune diseases. However, in order to rationally intervene to restore immune homeostasis, there is much that remains to be understood about the molecular phenotypes, mechanisms and pathways that govern the differentiation, expansion, maintenance, and regulatory function of CD8^+^ T_reg_. We have developed a unique model in which CD8^+^ regulatory T cells can be induced to suppress the development of autoimmune disease in an animal model of lupus, the (NZB/NZW) F1 (BWF1) mouse (3, 5, 25, 26). In this model, we have demonstrated that synthetic peptides (pCons) based on T cell determinants in the VH region of IgG which encode murine antibodies to DNA that bind to MHC Class I/II regions can activate CD8^+^ T cells *in vitro*, which can result in the suppression of co-cultured CD4^+^ T helper cells and B cell activities (26, 27). In addition, when pCons is administered *in vivo*, we can demonstrate the suppression of anti-DNA antibody production, and subsequent nephritis. However, the cellular and molecular phenotypes of pCons-induced CD8^+^ regulatory T cells are not yet completely clear. In this study, we have further provided novel information and defined the immunological and molecular phenotypes of pCons-induced CD8^+^ T regulatory cells and CD4^+^ regulatory T cells. We also showed that pCons treatment reduces apoptosis in CD4^+^ T cells, and CD8^+^ T cells and B220^+^ B cells. To the translational significance, we showed herein that pCons also induces CD8^+^FoxP3^+^ T_reg_ cells in healthy human peripheral blood mononuclear cells (PBMCs).

## Materials and Methods

### Mice

NZB (H-2d/d), NZW (H-2z/z) and NZB/NZW F1 (H-2d/z) mice were purchased from the Jackson Laboratories (Bar Harbor, ME, USA) or bred at the University of California Los Angeles (UCLA). All mice were treated in accordance with the guidelines of the University of California Los Angeles Animal Research Committee, an Institution accredited by the Association for Assessment and Accreditation of Laboratory Animal Care (AAALAC). Mice were housed in pathogen-free conditions according to the National Institutes of Health (NIH) guidelines for the use of experimental animals. Female mice were used for all experiments.

### Subjects

We enrolled 6 healthy female donors (19-70 years of age) with no history of autoimmune disease. Subjects had regular menstrual cycles and were not taking any contraceptives or sex hormones. Written informed consent was obtained from each subject who participated in the study. The study was approved by the Institutional Review Board (# 11-000907) of the University of California Los Angeles.

### Peptides

The pCons peptide used in this study and the MHC molecules they bind have been described earlier (26, 28). pCons (FIEWNKLRFRQGLEW), the artificial tolerizing peptide, contains T-cell determinants based on the J558 VH regions of several murine anti-dsDNA Ab from BWF1 mice (3, 5, 23, 26, 27, 29). Peptides were synthesized at Chiron Biochemicals (San Diego, CA, USA), purified to a single peak on high-performance liquid chromatography, and analyzed by mass spectroscopy for expected amino acid content.

### Treatment of Mice

Ten- to twelve-week-old female BWF1 mice received a single i.v. dose of 1 mg of pCons, dissolved in saline, as reported previously (26, 27, 30) for tolerance induction. For immunophenotyping of regulatory T cells, female BWF1 mice were used and injected with pCons. Control mice received either a similar amount of pNeg (negative control peptide) or saline.

### Cell Isolation, Preparation, Immunophenotyping, and Flow Cytometry

Spleen cells were isolated ~1 week after administration of the pCons peptide from tolerized, saline-treated, or naïve BWF1 mice. Single cell suspensions of splenocytes were prepared by passing cells through cell strainers (40µm) (Fisher). ACK lysing buffer, (Sigma, St Louis, MO, USA) was used to lyse red blood cells. Cells were washed and re-suspended in RPMI complete media. RPMI 1640-complete media was supplemented with L-glutamine (2 mM), penicillin (100 units/ml), streptomycin (0.1 mg/ml), 2-mercaptoethanol (Gibco) and 10% fetal bovine serum (FBS). FACS staining buffer was obtained from eBiosciences, BD Pharmingen, and/or BioLegend Inc. Cell subsets were further enriched following incubation with anti-CD4 (L3T4), anti-B (CD45R/B220), anti-CD8 (CD8a Ly-2), and microbeads from Miltenyi Biotech (Auburn, CA, USA). Purity of cells was determined to be more than 90% pure as assessed by flow cytometry (FACS). For immunophenotyping, isolated cells were washed with FACS buffer and 1–2 million cells were used for cell surface staining. Before staining, cells were incubated with rat anti-mouse CD16/CD32 (FC III/II receptor) Ab to block nonspecific binding.

For regulatory T cell immunophenotyping, spleen cells were stained with CD4 (L3T4), (RPA-T4), CD8 (Ly-2), CD25 (PC61), CD28 (37.51), CD62L (MEL-14), CD122 (TM-β1), PD1(RMP1), CCR7(4B12), GITR (DTA-1, AITR, TNFRSF18), CTLA-4 (UC10-4F10-11) and 4IBB(1AH2) antibodies for FACS analysis. Antibodies for cell surface staining and isotype controls were from BD Biosciences, BD Pharmingen, eBiosciences, or BioLegend. FoxP3 (PCH101) staining was performed with an eBiosciences intracellular kit (Cat #12-4776). Before intracellular FoxP3 staining, cells were stained with cell surface molecules (CD4, CD8, CD25, CD28, CD62L, CD122) as per manufacturer’s protocol. Cells were fixed and permeabilized, washed with permeabilization buffer, and then stained with anti-human FoxP3 (PCH101) antibody in 1X permeabilization buffer (eBiosciences), washed again with permeabilization buffer, and then the samples analyzed by FACS at the UCLA flow Core facility. Data was collected using an FACSCalibur (BD Biosciences) and analyzed with BD Cell Quest software (Becton-Dickinson, Mountain View, CA) or *De Novo* FCS Express Ver. 7 software (Ontario, Canada).

### Human Peripheral Blood Mononuclear Cells (PBMCs) Isolation and Preparation

For human studies, peripheral blood mononuclear cells (PBMCs) were isolated on a density gradient (Histopaque-1077, Sigma-Aldrich, St. Louis, MO, USA) from blood samples of healthy volunteers. Lymphocytes were washed twice in RPMI complete media. Red blood cells (RBC) were lysed with RBC lysing solution (Sigma-Aldrich, St. Louis, MO, USA). After washing cells were stained with fluorochrome -labeled monoclonal antibodies (mAbs) and analyzed by FACS.

### Western Blot Analysis

Western blot analyses were performed as described earlier (31). In brief, cell lysates were prepared from the CD8^+^ T cells of naïve and pCons-treated BWF1 mice. Cells were lysed with RIPA buffer (150 nM NaCl, 1.0% NP-40, 0.5% sodium deoxycholate, 0.1% SDS, 10 mM Tris, pH 7.3) supplemented with Protease Arrest protease inhibitor cocktail solution (G Biosciences, Maryland Heights, MO, USA). Protein was measured from each sample using the Bradford assay (Bio-Rad Laboratories, Hercules, CA, USA) and an equal amount of protein was loaded in each well. The lysates were resolved on a 4–12% NuPage gel (Invitrogen, Carlsbad, CA, USA) under reducing conditions. Proteins were electro-transferred onto a polyvinylidene fluoride membrane (Invitrogen). The membranes were blocked with 3% BSA and immunoblotted with a protein-specific antibodies [GPT2 (ab80947), Abcam; PD1 (DO-1), sc-126 Santa Cruz Biotechnology, Inc; PD1 (ab58811) Abcam; GADD45b (K-12), sc-133606, Santa Cruz Biotechnologies, Inc; p53 (DO-1) sc-126, Santa Cruz Biotechnologies, Inc, Santa Cruz, CA, USA, (1: 200 - 1:1000 dilution range); Bax (1:1000 dilution) Cat # #2772, Cell Signaling Technology, Danvers, MA; PDE3b, H-300, sc-20793 (1:1000 dilution); RGS16 (H-100), sc-30218 (1:1000 dilution) or β-actin (1:100 000 dilution; Sigma, Inc]. Following washing, the membranes were incubated in secondary antibodies (1:2500 dilution; Santa Cruz Inc, Santa Cruz, CA, USA). All blocking, incubation and washing steps were performed in TBST (TBS and 0.1% Tween-20). Proteins were visualized using ECL (GE Healthcare, Buckinghamshire, UK).

### RNA Isolation and Real-Time PCR

Total cellular RNA was isolated from purified cell subsets from saline-treated or pCons-tolerized BWF1 mice with TRIzol (Invitrogen, Carlsbad, CA, USA) as per manufacturer’s protocols. One-step-real time PCR was analyzed as described earlier (3, 5, 26, 29). Each experimental group consists of the pooled spleen cells of 3–4 mice from each group, naïve CD8^+^ T cells or tolerized CD8^+^ T cells. One-step RT-PCR was performed (Applied Biosystems, Foster City, CA, USA) using 100 ng of total RNA. Quantitative real-time reverse transcription was performed using TaqMan technology on an ABI Prism 7900 HT Sequence Detection System (Applied Biosystems). Primers and probes of regulator of G protein signaling genes (RGS2, RGS16, and RGS17), glutamic pyruvate transaminase 2 (GPT2), BAX (Bcl-2-associated X protein), programmed cell death-1 (PD1), growth arrest and DNA damage inducible 45 beta (GADD45β), and phosphodiesterase 3b (PDE3b), and GAPDH were obtained from Applied Biosystems (Foster City, CA, USA). The other oligonucleotide sequences used for the primers and TaqMan probes (Applied Biosystem, Foster City, CA) are described (3, 5, 26, 29). GAPDH was used as an endogenous control in each experimental set.

### Measurement of Apoptosis

Assays were performed to measure apoptosis as described earlier (5, 26). In brief, splenocytes were obtained from both naïve and pCons-treated BWF1 mice. RBC were lysed, cells washed, and stained with fluorochrome-labeled specific antibodies [CD4 (PerCP), CD8 (PE), B220 (APC), and Annexin V (FITC)] and flow cytometry performed.

### Statistical Analyses

Data was analyzed using GraphPad Prism 4.0 Software (San Diego, CA). Comparisons between the two groups were performed using paired one- or two-tailed Student’s *t*-test. Nonparametric testing among more than two groups was performed by one-way ANOVA. Results are expressed as mean ± SEM. p<0.05 was considered significant.

## Results

### pCons Treatment Alters Cell Surface Expression of CD25, CD122, and Increased Intracellular FoxP3 Expression in CD4^+^ T Cells and Further Modifies Cell Surface Expression of CD25, CD28, CTLA-4 and 41BB in CD8^+^ T Cells in BWF1 Mice

**Figure 1:**
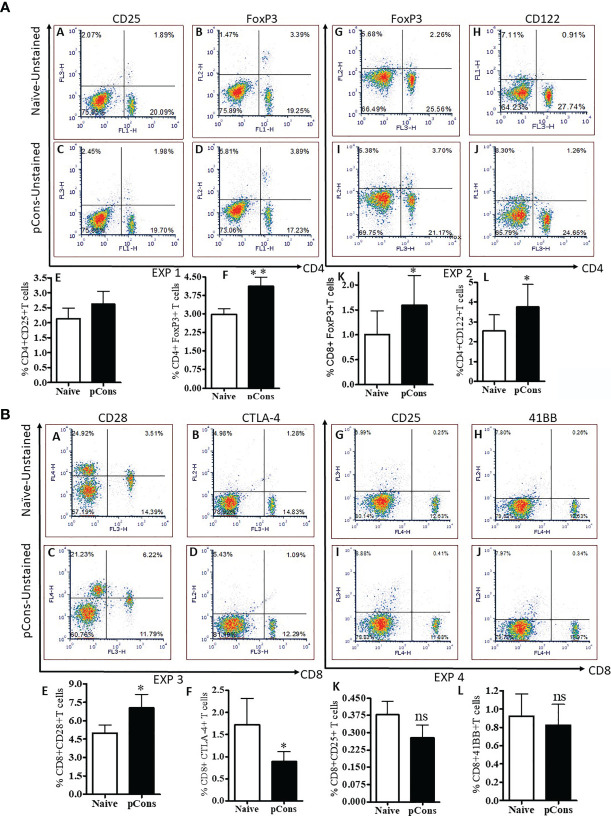
pCons treatment alters cell surface expression of CD25, CD122, and increased intracellular FoxP3 expression in CD4^+^ T cells and further modifies cell surface expression of CD25, CD28, CTLA-4 and 41BB in CD8^+^ T cells in BWF1 mice. Female 10-12-wk old BWF1 mice were treated with pCons (1 mg *i.v*.). After one-two week treatment, splenocytes were obtained both from naïve and pCons-treated BWF1 mice. RBC were lysed, cells washed, and stained with fluorochrome-labeled specific antibodies (CD4, CD8, CD25, CD28, CD122, CTLA-4, and 41BB). FACS analysis was performed on a FACSCalibur™ with cell Quest™ software (BD Biosciences, San Jose, CA) and analyzed using *De Novo* FCS Express software (Ontario, Canada). Intracellular FoxP3 expression was analyzed after cell fixation and permeabilization as per manufacturer’s protocol (eBiosciences, San Diego, CA, USA). **(A)** CD4^+^ T cells, and **(B)** CD8^+^ T cells data, two experiments each. **(A)** Exp 1. Panel (A) Naïve CD4^+^CD25^+^ T cells; (B) Naïve CD4^+^FoxP3^+^ T cells; (C) pCons CD4^+^CD25^+^ T cells; (D) pCons CD4^+^FoxP3^+^ T cells. (E) Cumulative data of CD4^+^CD25^+^T cells (4-5 experiments of two/three mice); (F) Cumulative data of CD4^+^FoxP3^+^ T cells (4-5 experiments of two/three mice). Exp 2. (G) Naïve CD4^+^FoxP3^+^ T cells; (H) Naïve CD4^+^CD122^+^ T cells; (I) pCons CD4^+^FoxP3^+^ T cells., (J) pCons CD4^+^CD122^+^ T cells. (K) Cumulative data (6 experiments) of CD8^+^FoxP3^+^ T cells. (L) Cumulative data of CD4^+^CD122^+^ T cells (4 experiments of two/three mice). **(B)** Exp 3. (A) Naïve CD8^+^CD28^+^ T cells; (B) Naïve CD8^+^CTLA-4^+^ T cells; (C) pCons CD8^+^CD28^+^ T cells; (D) pCons CD8^+^CTLA-4^+^ T cells. (E) Cumulative data of CD8^+^CD28^+^ T cells (5-6 experiments). (F) Cumulative data of CD8^+^CTLA-4^+^ T cells (5-6 experiments). Exp.4. (G) Naïve CD8^+^CD25^+^ T cells; (H) Naïve CD8^+^41BB^+^ T cells, (I) pCons CD8^+^CD25^+^ T cells; (J) pCons CD8^+^41BB^+^ T cells. (K) Cumulative (6-7 experiments data) of CD8^+^CD25^+^ T cells, (L) Cumulative (4 experiments data) of CD8^+^41BB^+^ T cells. Minimum 10,000 cells were gated, and only live cells were used for data analyses. Dead cells were excluded from the analyses. *p < 0.05. **p < 0. 001. ns, not significant.

To explore the tolerogenic immune responses of pCons peptide in the present study, we determined the various cell surface expression markers by flow cytometry in both CD4^+^ and CD8^+^ T cells in pCons-treated and negative control peptide and or saline-treated BWF1 mice. We found that pCons treatment increased the cell surface expression of CD25 and CD122 in CD4^+^ T cells compared to naïve CD4^+^ T cells (**Figure 1A** **Panels A, C, E, H, J, L**). We also found that pCons treatment increased the intracellular FoxP3 expression in pCons-treated CD4^+^ T cells (**Figure 1A** **Panels B, D, F, G, I, K**). Next, we investigated the effect of pCons on CD8^+^ T cells. Our data demonstrate that pCons treatment modified the cell surface expression of CD25 (increased), CD28 (increased), CTLA-4 (decreased) and 41BB (no changed) in CD8^+^ T cells (**Figure 1B** **Panels A–L**). Gating strategy is shown in (**Supplementary Figure 1A** and **Figure 1B** Panels A–H). Previously, we have demonstrated that pCons treatment increased the FoxP3 expression in CD8^+^ T cells (3, 26). In this study, we re-validated our previous findings of FoxP3 and added novel information for additional cell surface phenotypes including FoxP3 with cumulative data of 4-6 experiments (**Figure1A** **Panel K**). Altogether, these data indicate that pCons treatment induced the various cell surface markers including intracellular FoxP3 in both CD4^+^ and CD8^+^T cells.

### pCons Treatment Induces and Modifies Cell Surface Expression of CD62L, CD122, and CCR7 in CD8^+^ T Cells in BWF1 Mice

**Figure 2:**
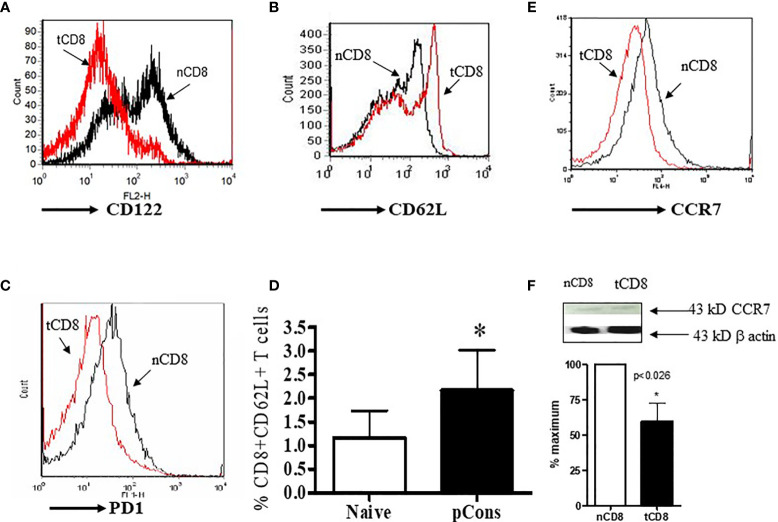
pCons treatment modified the cell surface expression of CD122, CD62L, CCR7, and PD1 in CD8^+^ T cells in BWF1 mice. Female 10-12-wk old BWF1 mice were treated with pCons (1 mg *i.v.*). After one-two week treatment, splenocytes were obtained both from naïve and pCons-treated BWF1 mice (2-3 mice in each group). RBC lysed, cells washed and stained with specific antibodies (CD8, CD62L, CD122, CCR7), and flow cytometry performed. **(A)** CD122 (PE) cell surface expression of naïve vs tolerized CD8^+^ T cells treated with pCons; **(B)** CD62L (FITC) cell surface expression of naïve vs tolerized CD8^+^ T cells pCons; **(C)** PD1 (PE) cell surface expression of naïve vs tolerized CD8^+^ (pCons); **(D)** Percent expression of naïve CD8^+^CD62L^+^ vs tolerized CD8^+^CD62L^+^ T cells (pCons); **(E)** CCR7 (APC) cell surface expression of naïve CD8^+^ vs tolerized CD8^+^ T cells (pCons-treated group). Minimum 10,000 cells were gated, and live cells were used for data analyses. Dead cells were excluded from the analyses. Data were analyzed with FCS Express Ver. 7 (*De Novo*, Ontario, Canada). *p < 0.05. **(F)** Naïve CD8^+^ and tolerized CD8^+^ T cells were obtained, lysed, and Western Blot analysis performed with CCR7 and β-actin antibodies. CCR7 value normalized to those of β−actin. *p < 0.05.

Previously we showed that pCons treatment increased the number of regulatory CD4^+^ and CD8^+^ T cells and modulated their functions including the ability for the suppression of anti-DNA antibody in BWF1 mice (3, 5, 23, 26). In the present study, we were interested to see whether pCons treatment of BWF1 mice changes the cell surface expression of CD122, CD62L, PD1, and CCR7 in CD8^+^ T cells since these markers have been implicated in the regulatory phenotypes of CD8^+^ T cells (32–36). We found two-fold increase in cell surface expression of CD62L and further increase in percent expression of CD8^+^ CD62L^+^ T cells and a decrease in the CD122 and CCR7 mean fluorescence intensity in pCons-treated CD8^+^ T cells compared to negative control peptide or saline-treated CD8^+^ T cells (**Figures 2A–E**). PD1 cell surface expression was decreased in pCons-treated CD8^+^ T cells (**Figure 2D**). Further, Western blot analysis demonstrated that protein levels of CCR7 are decreased in CD8^+^ T cells after pCons tolerance (**Figure 2F**). Taken together, these data suggest that pCons treatment has differential immune-regulatory effects on CD8^+^ T cells and on CD62L, CD122, PD1, and CCR7.

### pCons Treatment Modifies Expression of Regulator of G-Protein Signaling Genes (RGS2, RGS16, RGS17), Interferon-Induced, and Apoptotic Genes in CD8^+^ T Cells

**Figure 3:**
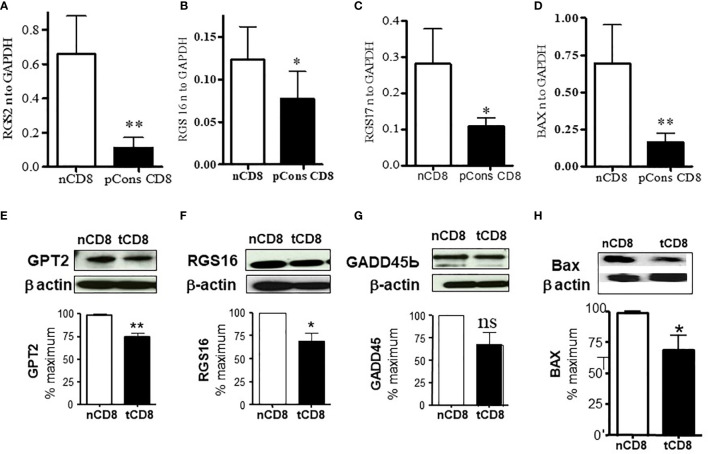
pCons treatment downregulated the expression of Regulator of G protein signaling (RGS2, RGS16, and RGS17) and Bax (Bcl2-associated X protein) in pCons-induced CD8^+^ T cells. Female 10-12-wk old BWF1 mice (n=3-5 mice) were treated with pCons (1 mg *i.v.*). After one-two week, splenocytes were obtained both from naïve and pCons-treated BWF1 mice. Naïve CD8^+^ T cells and pCons-treated CD8^+^ T cells were isolated from BWF1 mice spleen cells using microbeads from (Miltenyi Biotech (Auburn, CA, USA). Splenocytes were labelled with CD8-specific antibody, and naïve and pCons-treated CD8^+^ T cells sorted by FACS. Cells were lysed with RNA lysis solution (Trizol) and total cellular RNA obtained. Murine primers and probes (RGS2, RGS16, RGS17, BAX, and GAPDH) were obtained from Applied Biosystems (Foster City CA, USA). Real time PCR was performed with 100 ng of RNA with gene specific primers and probes comparing naïve and pCons-treated CD8^+^ T cells for each protein. **(A)** RGS2 normalized to GAPDH in naïve CD8^+^ T cells vs pCons-treated CD8^+^ T cells. **(B)** RGS16. **(C)** RGS17. **(D)** BAX. Data was normalized with GAPDH mRNA levels. **(E–H)**. Western blot analyses. **(E)** GPT2 protein level normalized to β-actin in naïve CD8^+^ T cells vs pCons-treated CD8^+^ T cells. **(F)** RGS16. **(G)** GADD45β. **(H)** BAX. Data was normalized to β actin protein levels. *p < 0.05, **p < 0.001. ns, not significant.

To determine whether there is cross-regulation of regulator of G protein signaling and interferon genes and whether pCons affect this cross-regulation in CD8^+^ T cells, we tested the expression of RGS and IFNs genes in pCons-treated CD8^+^ T cells. We have shown previously that pCons-induced CD8^+^ T regulatory cells are genetically reprogrammed following pCons induction (26, 29, 31). These pCons-induced CD8^+^ T_reg_ cells display i) resist to apoptosis; ii) have immunosuppressive programs; and iii) traffic to sites of inflammation to inhibit the development of autoantibody formation. Our previous gene chip array analyses demonstrated that pCons-induced CD8^+^ T_reg_ cells have upregulated genes including; interferon inducible *202b (Ifi202b), FoxP3, Bcl2*, transformation related protein *53 (TP53)* and interferon receptor *IFNAR1* (29). In the present study, we showed herein that pCons treatment significantly downregulated and decreased (~2-5 fold) the expression of 6 genes: *RGS2*, *RGS16*, *RGS17*, *BAX*, *GPT2*, *GADD45β* (**Figures 3A–D**). We further confirmed the downregulation of GPT2, RGS16, GADD45β, and BAX proteins by Western blot analyses (**Figures 3E–H**). The downregulated BAX expression (37) in pCons-induced CD8^+^ T cells may contribute to the survival of these cells *in vitro*/*vivo*. Overall, these data indicate that pCons regulates RGS, IFNs, and apoptotic genes.

### pCons Treatment Reduces Apoptosis of CD4^+^ and CD8^+^ T Cells, and of B220^+^ B Cells in BWF1 Lupus Mice

**Figure 4:**
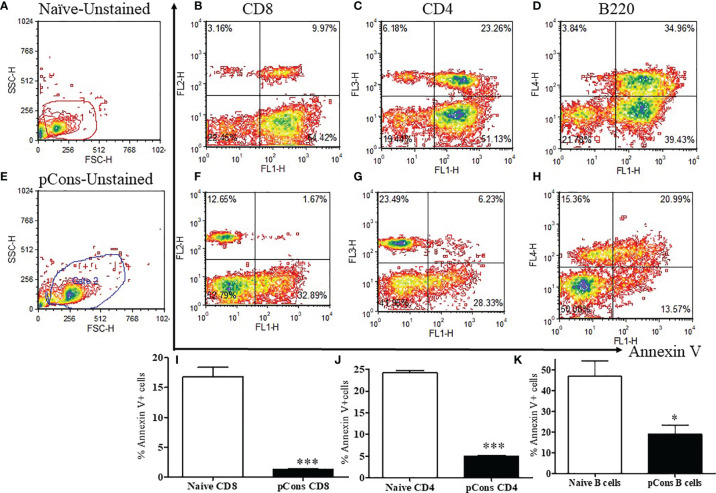
pCons treatment alters and reduces apoptosis in both T and B cells of BWF1 mice. Female 10-12-wk old BWF1 mice were treated with pCons (1 mg *i.v*.). After one-two week treatment, splenocytes were obtained from both naïve and pCons-treated BWF1 mice (two/three mice in each group). RBC were lysed, cells washed, and stained with fluorochrome-labeled specific antibodies [CD4 (PerCP), CD8 (PE), B220 (APC), and Annexin V (FITC)] and flow cytometry performed. **(A)** Naïve unstained splenocytes; **(B)** Naïve CD8^+^ T cells; **(C)** Naïve CD4^+^ T cells; **(D)** Naïve B220^+^ B cells; **(E)** pCons-treated unstained splenocytes; **(F)** pCons-treated CD8^+^ T cells, **(G)** pCons-treated CD4^+^ T cells; **(H)** pCons-treated B220^+^ B cells; **(I)** Combined data of experiments (n=3-4) of naïve vs pCons-treated CD8^+^Annexin V^+^ T cells; **(J)** Combined data of experiments (n=3-4) of naïve vs pCons-treated CD4^+^Annexin V^+^ T cells; **(K)** Combined data of experiments (n=3-4) of naïve vs pCons-treated B220^+^Annexin V^+^ B cells. Minimum of 10,000 live cells were gated for data analyses. Dead cells were excluded from the analyses. Data was analyzed with FCS Express Ver. 7 (*De Novo*, Ontario, Canada). Statistical differences were determined by paired two-tailed Student’s t-test. *p < 0.05, ***p < 0.0001.

Since earlier studies demonstrated that apoptosis affects immune tolerance, we were interested to investigate whether pCons influences apoptosis in various immune cell subsets including T cells which play an important role in lupus. To address this, we treated BWF1 lupus mice with pCons. After 1-2 weeks, splenocytes were obtained, stained with Annexin V FITC, and then stained with fluorochrome-labeled CD4^+^, CD8^+^ and B cells specific monoclonal antibodies, and analyzed by FACS. We found that pCons treatment significantly decreased (~5 fold) apoptosis in CD4^+^ T cells, CD8^+^ T cells (8-10-fold) and B220^+^B cells (~2.5-fold) compared to naïve or saline treated cells (**Figures 4A–K**). These data clearly demonstrate that pCons reduces apoptosis in both T and B cells.

### pCons Increases CD8^+^FoxP3^+^ T Cells in Healthy Human Subjects

**Figure 5:**
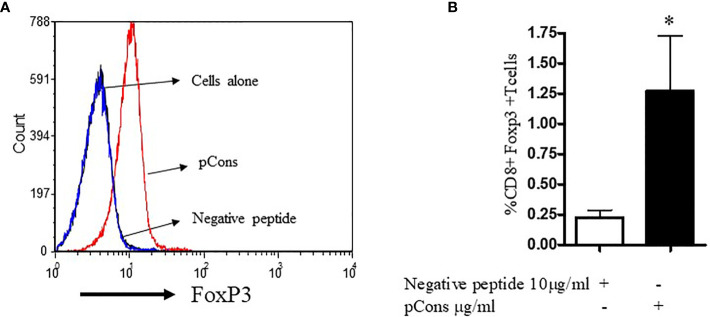
pCons treatment increases CD8^+^FoxP3^+^ T cells in healthy subjects. 10x10^6^ peripheral blood mononuclear cells (PBMCs) were obtained from healthy volunteers (n=6). Cells were cultured with recombinant IL-2 (50 U/ml), negative control peptide A or pCons peptide (10 ug/ml) for 24-48 hours. Cells were stained with CD4, CD8, CD25 and FoxP3 fluorochrome-conjugated antibodies. FoxP3 intracellular staining was performed after cell surface staining. Cell were fixed and permeabilized as per manufacturer’s protocol. Minimum 10,000 cells were gated, and only live cells were used for data analyses. Dead cells were excluded from the analyses. **(A)** Mean fluorescence intensity (MFI) of FoxP3 among naïve, negative control peptide, and pCons-treated PBMCs of healthy subjects. **(B)** Percent expression of CD8^+^FoxP3^+^ T cells between negative control peptide and pCons-treated PBMCs of healthy controls. Dead cells were excluded from the analyses. Data were analyzed with FCS Express Ver. 7 (*De Novo*, Ontario, Canada) *p < 0.05.

Having examined the immunomodulatory properties of pCons in murine cells, we investigated whether pCons induces CD8^+^FoxP3^+^ T regulatory cells in healthy human subjects. To determine this, we isolated PBMCs from healthy subjects and cultured them with negative control peptide (pNeg) and pCons peptide (10 μg/ml) for 24-48 hours. After culture, cells were washed, stained with CD4, CD8, CD25 and FoxP3 fluorochrome-labeled monoclonal antibodies and analyzed by flow cytometry. As shown in **Figure 5**, pCons significantly increases the mean fluorescence intensity of cells expressing FoxP3 (**Figure 5A**) and the percentage of CD8^+^FoxP3^+^ T cells are significantly increased (~5 fold) (**Figure 5B**). These data suggest that pCons induces CD8^+^FoxP3^+^ T_reg_ cells and has translational significance in humans.

## Discussion

Regulatory T cells play a key role in the regulation of immune responses and maintaining immune homeostasis. Impairment in the development and function of regulatory T cells is a major contributing factor in the development of autoimmune diseases, including SLE (17, 19, 38–40). Thus, inducing and regulating the function of T_reg_ is currently one of the prime goals not only in the study of autoimmune diseases, but graft versus host disease, organ transplantation, and neoplastic disease (41–43). In this study, we provided novel information for the immunological, cellular, and molecular phenotypes of regulatory T cells especially CD8^+^ T_reg_ cells induced by the pCons treatment in BWF1 lupus mice. pCons-induced CD8^+^ T cells express high levels of CD62L, and low levels of CD122, PD1, CTLA4, and CCR7. We did not find major changes in the expression of 41BB. Further, pCons modulated the expression of CD25, CD28, CD122, and FoxP3 in both CD4^+^ and CD8^+^ T_reg_ cells. The molecular phenotypes of suppressive CD8^+^ T_reg_ cells include low level of regulator of G protein signaling *(RGS2, RGS16, RGS17), BAX, GPT2, GADD45β, PDE3b*, and *PD1* (programmed cell death 1). The downregulation of these genes in pCons-tolerized CD8^+^ T cells was confirmed with Western blot analyses (**Figure 3**). Thus, our data revealed a molecular signature phenotype in CD8^+^ T cells induced by pCons peptide in BWF1 lupus mice that has clinical and functional importance in the immune tolerance and their immunoregulation. For example, L-selectin (CD62L) is a type-I transmembrane glycoprotein and adhesion molecule that plays an important role in T cell activation. A recent study revealed that CD62L expression on blood basophils may predict future response to standard induction therapy for patients with lupus nephritis (44). In addition, another study found that the expression levels of CD62L decreased on T cells during the inflammatory state and levels of CD8^+^CD62L^+^ T cells negatively correlated with disease severity (45). Previously glucocorticoids have been shown to increase the CD62L expression in patients with lupus (46). In agreement with our study, a recent study also showed an increase in expression of CD62L on CD8^+^ regulatory T cells in lupus mice (47). Thus, altogether, our finding of increased CD62L expression levels on CD8^+^ T cells after induction by pCons treatment in BWF1 lupus mice points to a therapeutic beneficial effect.

Recent evidence suggest that both CD8^+^CD122^+^ and CD8^+^C122^-^ T cells are regulatory and can suppress autoimmunity (7, 48). Importantly, these cells express CD122 (IL-2Rβ), CD62L^high^, PD1^low^ and CCR7^low^ (32, 49). Further, our data showed that pCons-induced CD8^+^ T cells in BWF1 mice have less cytotoxic-T lymphocyte-antigen-4 (CTLA-4) expression as compared to naïve CD8^+^ T cells. This is an important finding, since CTLA-4 (CD152) is an inhibitory cell-surface molecule that plays an important role in the promotion of anergy, immune regulation, and the prevention of autoimmunity. Abnormal function and susceptibility of CTLA-4 gene expression has been reported in SLE patients (50, 51). Further, it was demonstrated that CTLA-4 modulates regulatory and follicular helper T cells, thus controlling humoral immunity (52–54). CTLA-4 has been shown to downregulates CD80 and CD86 on antigen presenting cells (APC) (54). However, its precise mechanism of action has not been fully understood. Thus, our data supports the notion that pCons-induced CD8^+^ T cells are regulatory in nature and possess all the cellular and immunological phenotypes to induce immune tolerance.

Recent reports suggest that CCR7 was involved in the progression of lupus and its expression was increased in SLE patients (55, 56). Additionally, CCR7-CCL19 couples interaction of T helper, and B cells, and dendritic cell migration (56); thus CCR7 helps in immune complex deposition and autoantibody production. Our findings of reduction of cell surface expression of CCR7 in CD8^+^ T cells is important because CCR7, a G protein-coupled receptor may also help in production of TGFβ. We have previously shown that pCons-induced CD8^+^ T cells increase TGFβ mRNA and protein levels (3, 5, 26, 31); therefore, it is possible that *TGF*β may be released *via* exosomes in CD8^+^ T cells in a CCR7-dependent manner. Exosomes are important in immunity (57), and we envision that they may play a role in CD8^+^ T_reg_-mediated suppression to establish and maintain self-tolerance. Earlier, we found that the mRNA of CCR7 was increased in pCons-tolerized CD8^+^ T cells (29). These differences may be due to cell surface trafficking and or differences in transcription/translational or “half-life” of CCR7 after pCons treatment in our model system. It is also conceivable that upregulated genes and cell surface receptors, e.g., IFNAR, IFI202b, may facilitate the expression, packaging, and release of TGFβ. In contrast, downregulation of CCR7 signaling may halt the inflammatory signals in pathogenic effectors CD4^+^ T cells, dendritic cells, antigen presenting cells (APCs), and B cells. Thus, CCR7 plays an important synergistic role in our model of immune tolerance. However, detailed mechanistic studies will be needed to address this issue.

Our findings that pCons-induced CD8^+^ T cells have decreased level of RGS proteins are important because reduction of RGS2 signaling increases Ca^2+^ mobilization and ERK1/2 activation in response to GPCR stimulation (58) which may be contributory to the observed CD8^+^ T_reg_ expansion and maintenance. RGS proteins are potent GTPase-activating proteins (GAP) for heterotrimeric G protein (G_q_, G_i_, and G_o_ family) alpha subunits acting as multifunctional inhibitors of signal transduction in many cells (59, 60), including “fine-tuning” GPCR signaling in lymphocytes (61). In particular, *RGS2*, also known as growth-inhibitory protein, plays a role in leukemogenesis (62). Thus, our finding that multiple RGS proteins are downregulated in tolerized CD8^+^ T cells (**Figure 3**) reinforces the positive effect on GPCR signaling pathways, together with reduced PDE3b, that may enhance cAMP signaling and plays an important role in the suppression of T cell function (63). The downregulation of PDE3b has been associated with enhanced insulin secretion, suggesting that secretion of other factors could also be positively modulated. Consistent with the notion that reversal of its cAMP-degrading activity is important for maintenance of CD8^+^ T regulatory cells. However, future studies will be required to address these possibilities.

In the current study, we found that GPT2 protein level was significantly decreased following pCons treatment in CD8^+^ T cells in BWF1 mice. GPT2 (glutamic-pyruvic transaminase 2) also known as alanine aminotransferase (ALT) plays an important role in gluconeogenesis and amino acid homeostasis, and is an hepatic enzyme/biomarker (64) that is upregulated in disease states. GPT2 has been shown to exacerbating autoimmune disease (65), and its levels were shown to be increased in NZB/NZW F1 mice (66). Increased serum alanine aminotransferases have been reported to be associated with anti-mitochondrial antibodies in SLE patients with autoimmune liver disease (67). Further Liu *et al.* found that liver injury including increased level of ALT correlates with biomarkers of autoimmunity and disease activity in patients with SLE (68). Thus, our finding that pCons treatment reduces GPT2 protein level has both clinical and translational significance in immune tolerance of our model and in SLE.

Previously, we demonstrated that pCons-induced CD8^+^ regulatory cells have upregulated genes including interferon inducible *202b (Ifi202b), FoxP3, Bcl2*, transformation related protein *53 (TP53)* and interferon receptor *IFNAR1* (29). We showed previously utilizing gene silencing studies that these genes are important in the suppression of anti-DNA ab in the BWF1 lupus mice (3, 5, 26, 31). In the current study, we added novel information of candidate’s downregulated genes in CD8^+^ T cells. For example, the *Bax* gene has been implicated in lupus nephritis and in apoptosis (37) suggesting that apoptosis dysregulation in SLE was affected by polymorphic variants in apoptotic-related genes including *Fas, FasL, Bcl2*, and *Bax*. While high expression of *FasL* expression contributes to increased apoptosis and to the breakdown of immune tolerance favoring autoantibody production and inflammation, low expression of the Bax protein was found to be protective in the SLE patients (69). In general, apoptotic T cells and neutrophils are increased in SLE patients and have positive correlation with SLE disease activity index (70, 71). Earlier, we showed that apoptosis was decreased in pCons-induced CD8^+^ T cells (26). In this study, we also found decreased percent of annexin V^+^ T cells and B cells in pCons-treated BWF1 mice (**Figure 4**). We postulate that pCons treatment has direct effect on both T and B cells. In addition, pCons might have an indirect effect through CD8^+^ T_regs_. This would require additional experiments to pinpoint the exact role. Thus, our finding of reduced Bax and annexin V in pCons-induced CD8^+^ T cells agrees with previous studies and suggests clinical significance. Similarly, GADD45β is a critical regulator of autoimmunity (72) that plays an important role in B cell apoptosis in response to *Fas* stimulation through activation of NF-κB (73). A recent report suggests that ablation of GADD45β ameliorates the inflammation and renal fibrosis caused by unilateral ureteral obstruction (UUO) in a chronic kidney disease mouse model (74). Previously it was shown that GADD45β was also induced in CD4^+^ T cells by inflammatory cytokines, such as IL-12 and IL-18 (75, 76). Furthermore, mRNA expression of GADD45β was associated with cytokine production and T helper cell differentiation (77, 78) and a genetic polymorphism study indicated a role for GADD45β in rheumatoid arthritis and lupus (79). Based on all these data, its plausible to hypothesize that some of the downregulated genes in pCons-induced CD8^+^ T_reg_ cells may be regulated by specific miRNAs as these molecules have been identified with important roles in immune regulation (80). How these genes or their gene products cross-regulate in the overall suppression mechanism in our immune tolerance model has not been fully elucidated. Future detailed mechanistic studies are warranted to pinpoint the exact role.

In addition, we have shown herein that pCons peptide induces CD8^+^FoxP3^+^T_reg_ cells in healthy human subjects suggesting translational significance. This finding is significant since patients with SLE have circulating T cells that can be activated by various peptides isolated from the variable regions of human anti-DNA antibodies (81, 82). Although, we were not able to study the modulation of CD8^+^FoxP3^+^T_regs_ in SLE patients with pCons, it may be possible that the pCons-modulation of CD8^+^ T_regs_ can be employed to reset the regulatory function of CD8^+^ regulatory T cells in lupus patients. Future study will be required to address this issue.

In summary, we found that pCons treatment promoted tolerogenic immune responses and modified the various cellular and molecular phenotypes in both CD8^+^ and CD4^+^ T regulatory cells in BWF1 mice. The data further demonstrate that CD8^+^FoxP3^+^ T cells can be modulated by pCons peptide in human cells indicating clinical and translational significance in SLE.

## Data Availability Statement

The raw data supporting the conclusions of this article will be made available by the authors, without undue reservation.

## Ethics Statement

The animal study was reviewed and approved by the Chancellor Animal Review Committee (ARC), University of California, Los Angeles. The human study was reviewed and approved by the University of California, Los Angeles, Institutional Review Board (UCLA-IRB).

## Author Contributions

RPS contributed to the experimental design, obtaining funding, conducting experiments, analyzing data, preparing figures, and writing of the manuscript. BHH contributed to funding and editing of the manuscript. DSB contributed to figure and manuscript editing. All authors contributed to the article and approved the submitted version.

## Funding

This work was supported by the NIH grants AR54034, AI 083894, AI65645 to RPS; UCLA Senate Core Grant to BHH and RPS; UCLA Oppenheimer Clinical Seed Grant and American Autoimmune Related Disease Association grant to RPS.

## Conflict of Interest

The authors declare that the research was conducted in the absence of any commercial or financial relationships that could be construed as a potential conflict of interest.

## Publisher’s Note

All claims expressed in this article are solely those of the authors and do not necessarily represent those of their affiliated organizations, or those of the publisher, the editors and the reviewers. Any product that may be evaluated in this article, or claim that may be made by its manufacturer, is not guaranteed or endorsed by the publisher.

## Supplementary Material

Supplementary Figure 1
**(A, B)** Splenocytes were obtained both from naïve and pCons-treated BWF1 mice. RBC were lysed, cells washed, and stained with fluorochrome-labeled specific antibodies (CD4, CD8, CD25, CD28, CD122, CTLA-4, and 41BB). Live cell gating strategy is shown in **(A)** (panels A–D) for CD4^+^ T cells and **(B)** (panels E-H) for CD8^+^ T cells. Lymphocytes were first identified by a low forward scatter (FSC) and low side scatter (SSC) gate, and then further phenotyped for CD4 (CD4), CD8 (CD8a) and B (B220) cells, followed by gating for CD25, CD28, CTLA-4, 41BB, CD122. Intracellular FoxP3 expression was analyzed after cell fixation and permeabilization as per manufacturer’s protocol (eBiosciences, San Diego, CA, USA).
