# Supplementary figures and images for: Cellular and Molecular Phenotypes of pConsensus Peptide (pCons) Induced CD8+ and CD4+ Regulatory T Cells in Lupus
Source: Front Immunol. 2021 Nov 19;12:718359. doi: 10.3389/fimmu.2021.718359 (PMC8640085; doi:10.3389/fimmu.2021.718359)

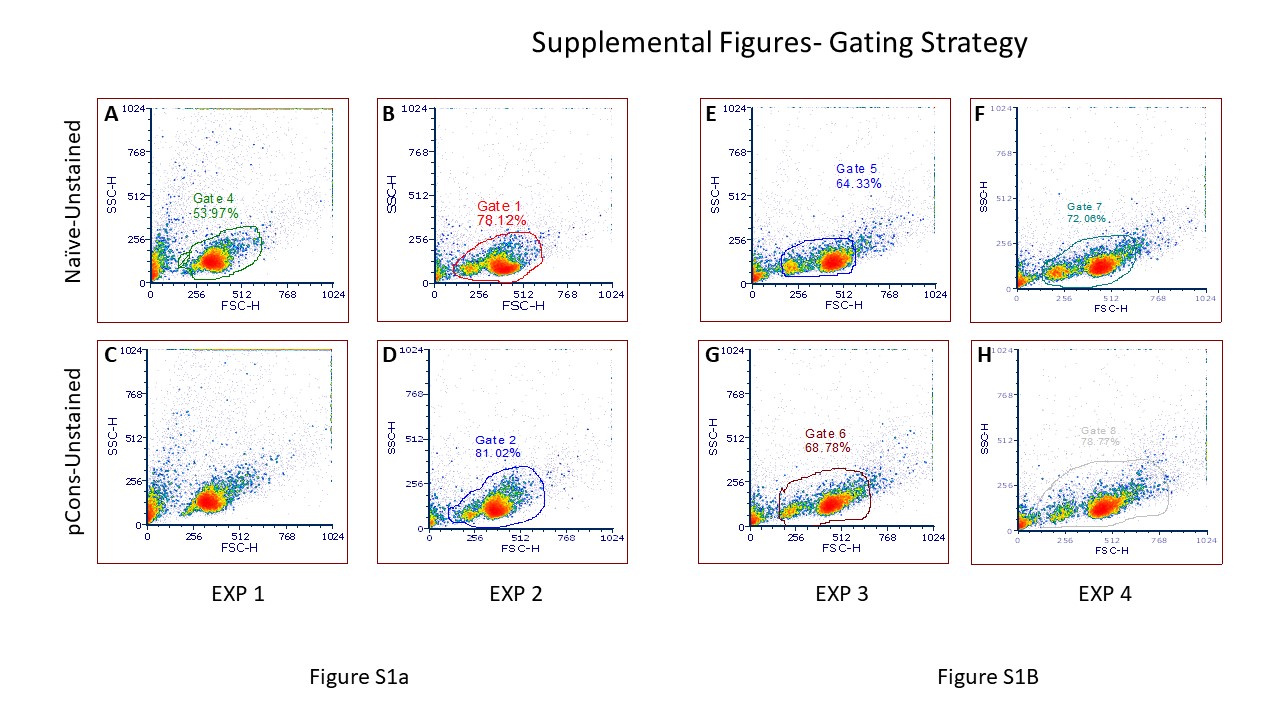

Supplement: Supplementary Figure 1 — (A, B) Splenocytes were obtained both from naïve and pCons-treated BWF1 mice. RBC were lysed, cells washed, and stained with fluorochrome-labeled specific antibodies (CD4, CD8, CD25, CD28, CD122, CTLA-4, and 41BB). Live cell gating strategy is shown in (A) (panels A–D) for CD4+ T cells and (B) (panels E-H) for CD8+ T cells. Lymphocytes were first identified by a low forward scatter (FSC) and low side scatter (SSC) gate, and then further phenotyped for CD4 (CD4), CD8 (CD8a) and B (B220) cells, followed by gating for CD25, CD28, CTLA-4, 41BB, CD122. Intracellular FoxP3 expression was analyzed after cell fixation and permeabilization as per manufacturer’s protocol (eBiosciences, San Diego, CA, USA). [file Image_1.jpeg]
